# Congenital Long QT Syndrome: From Conventional Management to Emerging Biological Therapies

**DOI:** 10.31083/RCM51843

**Published:** 2026-09-07

**Authors:** Qing Liu, Yifei Wang, Ping Zhang, Tingting Lv

**Affiliations:** ^1^Department of General Practice, Suining Central Hospital, 629000 Suining, Sichuan, China; ^2^Clinical Medical College, Qinghai University, 810000 Xining, Qinghai, China; ^3^Department of Cardiology, Beijing Tsinghua Changgung Hospital, School of Clinical Medicine, Tsinghua University, 102218 Beijing, China

**Keywords:** long QT syndrome, ion channelopathy, β-blockers, biological therapy, gene editing

## Abstract

Congenital long QT syndrome (LQTS), the first identified cardiac ion channelopathy, is characterized by delayed ventricular repolarization and a prolonged heart rate-corrected QT interval (QTc), conferring an increased risk of malignant ventricular arrhythmias and sudden cardiac death. Current standard-of-care management for LQTS, including beta-blockers, sodium channel blockers, left cardiac sympathetic denervation (LCSD), and implantable cardioverter-defibrillators (ICDs), reduces adverse events and improves survival but primarily mitigates downstream electrophysiological consequences rather than correcting the underlying genetic defects. Rapid advances in molecular biology have driven the development of mechanism-directed strategies such as gene editing, RNA interference (RNAi), and experimental antibody-based interventions, which aim to restore channel function or modulate channel regulation at the source. This review synthesizes current knowledge on LQTS classification and pathogenesis and evaluates both guideline-directed therapies and emerging molecular interventions. We emphasize subtype-specific approaches, highlight the rationale and preclinical progress for gene- and RNA-based therapies, and outline practical considerations for translation, including delivery, durability, and safety. Therefore, this review provides a comprehensive, mechanism-based framework to inform future precision medicine approaches for LQTS.

## 1. Introduction

Congenital long QT syndrome (LQTS) is a rare hereditary ion channelopathy characterized by abnormal ventricular repolarization, most notably a prolonged heart rate-corrected QT interval (QTc) observed on a resting electrocardiogram (ECG). Clinically, LQTS presents as syncope, seizures, or sudden cardiac death despite normal cardiac structure [1,2]. The hallmark life-threatening ventricular arrhythmia in LQTS is torsades de pointes (TdP), a polymorphic ventricular tachycardia characterized by a progressive twisting of the QRS axis around the isoelectric baseline. TdP is often initiated by a short-long-short sequence and may degenerate into ventricular fibrillation (VF) if sustained, resulting in hemodynamic collapse and sudden cardiac death. Although other forms of polymorphic ventricular tachycardia may also occur, TdP remains the most classically arrhythmia associated with QT prolongation (Fig. 1). The estimated incidence is approximately 1/2000 to 1/2500 individuals [3,4], with a slightly higher prevalence in females compared to males. Notably, the subtypes LQT1, LQT2, and LQT3 account for over 90% of cases [5]. The marked genetic heterogeneity of LQTS contributes to diverse clinical phenotypes and trigger profiles, thereby complicating risk stratification and therapeutic decision-making.

**Fig. 1.**
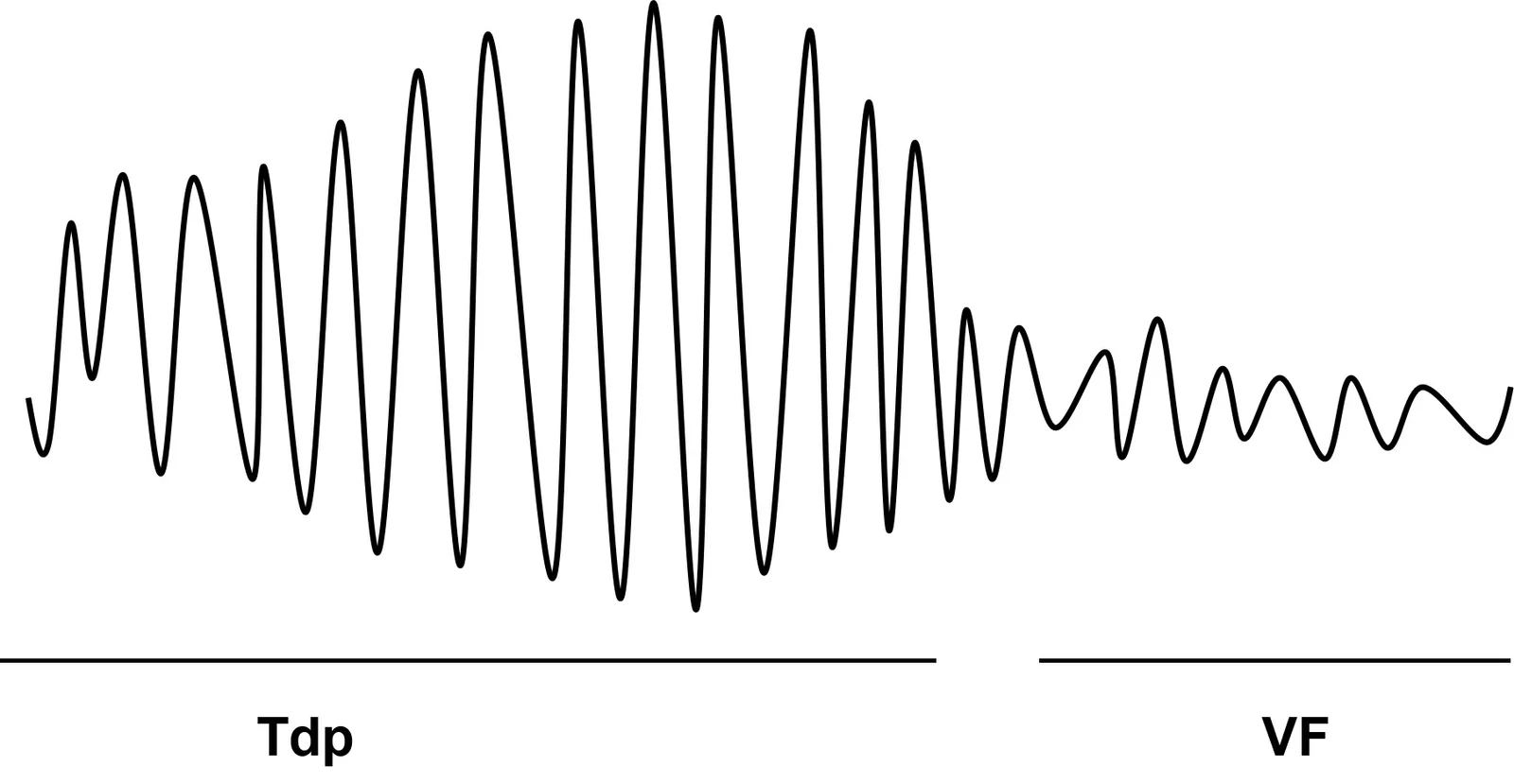
**Schematic ECG tracing illustrating torsades de pointes (TdP) degenerating into ventricular fibrillation (VF) in congenital long QT syndrome**. TdP is characterized by the typical twisting of the QRS complexes around the isoelectric baseline. VF represents a disorganized ventricular rhythm that may result from sustained TdP. ECG, electrocardiogram.

Conventional treatment approaches for LQTS can be categorized according to their primary therapeutic role. Preventive strategies, such as β-blockers and left cardiac sympathetic denervation (LCSD), aim to reduce the occurrence of ventricular arrhythmias by attenuating adrenergic triggers and reducing sympathetic input [6,7]. In contrast, implantable cardioverter-defibrillators (ICDs) serve primarily as rescue therapies that terminate life-threatening ventricular arrhythmias once they occur, but do not prevent their initiation [8]. Although these approaches reduce arrhythmia-related morbidity and mortality, they do not eliminate the residual arrhythmic risk, particularly in high-risk patients. In addition, patients may encounter challenges such as drug intolerance, diminished quality of life, and treatment-related complications, including infection, lead dislocation, and vascular issues, particularly among younger patients [9]. Moreover, these treatments do not address the underlying molecular defects [10]. Consequently, there is a pressing need to develop more precise and mechanism-based therapeutic strategies for LQTS. In 1972, Friedmann and Roblin introduced the hypothesis that gene therapy could be utilized in the treatment of human diseases [11]. With the rapid advancements in genetic engineering and molecular biology, research into ion channel disorders has become increasingly sophisticated, and biological therapies such as gene editing, RNA interference, and antibody therapy have emerged as focal points of research [12,13,14]. However, most of these approaches remain in the preclinical stage. Therefore, it is critical to both assess the current clinical treatment landscape and explore the potential of innovative molecular therapies. This review aims to systematically examine existing therapies and to evaluate emerging biological strategies for LQTS.

## 2. Classification of LQTS

Long QT syndrome is classified into two main categories: congenital long QT syndrome and acquired long QT syndrome, based on the presence or absence of identifiable secondary triggers. A previous study has suggested that mutations in 17 genes are associated with LQTS [15]. However, recent research has revealed that only 3 genes (*KCNQ1*, *KCNH2*, and *SCN5A*) are currently considered to have definitive evidence for typical congenital LQTS. The genes *CALM1*, *CALM2*, *CALM3*, and *TRDN* are considered to provide strong or clear evidence of pathogenicity for LQTS with atypical characteristics [16]. Acquired LQTS typically arises from the use of specific medications (such as antiarrhythmic drugs, antibiotics, and antipsychotic drugs), electrolyte imbalances (including hypokalemia and hypomagnesemia), and certain medical conditions (such as hypothyroidism) [17,18]. This review primarily focuses on the clinical characteristics and treatment implications of the most prevalent subtypes of congenital LQTS.

Table 1 lists genes with definitive or strong gene-disease clinical validity for congenital LQTS as determined by the ClinGen curation framework and the international reappraisal [16,19].

**Table 1.** **Genetic evidence for genes associated with congenital LQTS**.

Gene	Locus	Protein	Functional effect	Evidence level (ClinGen)	Frequency (%)
*KCNQ1*	11p15.5	K_v_7.1	Reduced I_Ks_	Definitive	30%–35%
*KCNH2*	7q36.1	hERG	Reduced I_Kr_	Definitive	30%–45%
*SCN5A*	3p22.2	Na_v_1.5	Increased I_Na,L_	Definitive	5%–10%
*CALM1*	14q32.11	Calmodulin-1	Abnormal Ca^2+^ signaling	Definitive^*^	Rare
*CALM2*	2p21	Calmodulin-2	Abnormal Ca^2+^ signaling	Definitive^*^	Rare
*CALM3*	19q13.32	Calmodulin-3	Abnormal Ca^2+^ signaling	Definitive^*^	Rare
*TRDN*	6q22.31	Triadin	N/A	Strong^#^	Rare

^*^ LQTS that manifests during infancy or early childhood, characterized by heart block and marked QT prolongation. ^#^ In early childhood, QT prolongation, negative T waves in precordial leads, and exercise-induced arrhythmias are associated with homozygous or compound heterozygous frameshift mutations. I_Ks_, slow delayed rectifier potassium current; I_Kr_, rapid delayed rectifier potassium current; I_Na,L_, late sodium current.

### 2.1 Long QT Syndrome Type 1(LQT1)

LQT1 accounts for approximately 30% to 35% of all genotyped LQTS cases [20,21]. The disease-causing gene, *KCNQ1*, encodes the α-subunit of the voltage-gated potassium channel K_v_7.1 (Fig. 2). This subunit partners with the auxiliary β-subunit *KCNE1* to form a functional channel that mediates the slowly activating delayed rectifier potassium current (I_Ks_) [22]. This current is activated in response to sympathetic stimulation. Mutations in the *KCNQ1* gene reduce I_Ks_ current density. In individuals without LQT1, sympathetic activation enhances I_Ks_ channel function, thereby accelerating ventricular repolarization and shortening the QT interval [23]. However, in LQT1 patients, the QT interval fails to shorten with increasing heart rate, leading to QT prolongation during exercise or emotional stress [24]. As a result, individuals with LQT1 are particularly susceptible to arrhythmic events during physical exertion. Notably, gain-of-function mutations in *KCNQ1* can cause short QT syndrome type 2 (SQT2), contrasting with the loss-of-function mechanism in LQT1. The R259H mutation increases I_Ks_ current density by approximately 3-fold through markedly slowing channel deactivation, thereby shortening the QT interval [25].

**Fig. 2.**
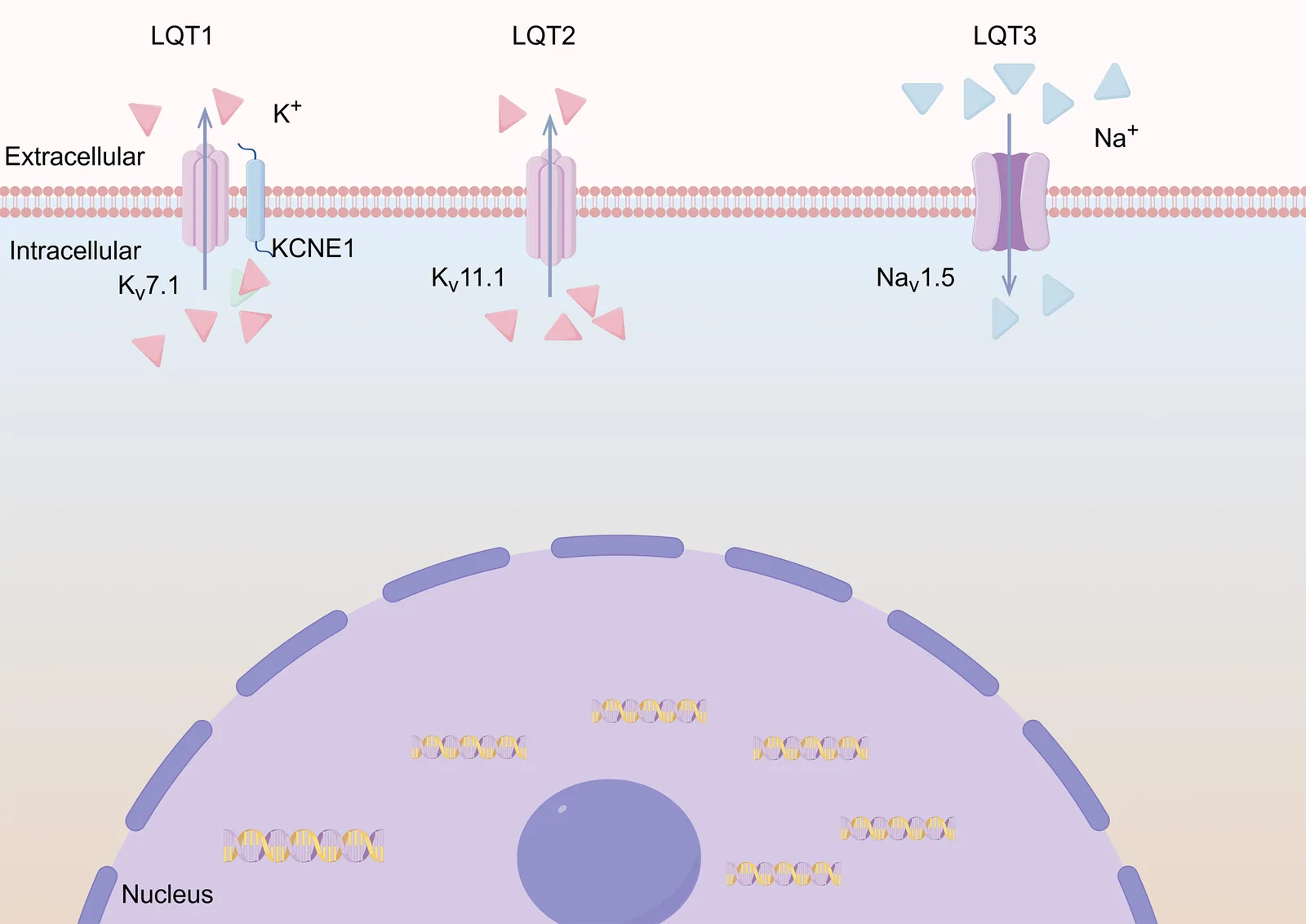
**Common channel diagrams related to long QT syndrome**. Pathogenic variants in *KCNQ1*, which encodes the α-subunit of the voltage-gated potassium channel K_v_7.1, result in LQT1. The *KCNH2* gene encodes the voltage-gated potassium channel K_v_11.1, resulting in LQT2. The *SCN5A* gene encodes the Na_v_1.5 α -subunit of the cardiac sodium channel, resulting in LQT3. The pink triangle represents K^+^, and the blue triangle represents Na^+^.

### 2.2 Long QT Syndrome Type 2(LQT2) 

LQT2 represents one of the most common genotyped subtypes of LQTS, accounting for approximately 30% to 45% of cases [26]. It is primarily associated with mutations in the *KCNH2* gene, also known as hERG. This gene encodes the voltage-gated potassium channel K_v_11.1, which conducts the rapid delayed rectifier potassium current (I_Kr_) [27,28]. Loss-of-function *KCNH2* (hERG) variants commonly cause reduced I_Kr_ through impaired channel trafficking, resulting in endoplasmic reticulum (ER) retention and enhanced degradation via proteasome or autophagy pathways [29]. For example, the expression of the G604S-hERG variant in HEK293 cells demonstrated that the mutant channel was retained in the endoplasmic reticulum, triggered ER stress, and underwent ubiquitin-proteasome-mediated degradation, ultimately leading to a marked reduction in the I_Kr_ current [30]. Clinically, patients with LQT2 often experience ventricular arrhythmias triggered by emotional stress or sudden auditory stimuli—such as alarms or ringing phones—especially in quiet environments. These events may result in sudden syncope or sudden cardiac death [31,32]. Furthermore, a previous study has shown that LQT2 patients with mutations located in the transmembrane pore region of *KCNH2* have significantly longer heart rate–corrected QT (QTc) intervals, more severe clinical symptoms, and an earlier onset of arrhythmia-related cardiac events compared to those with mutations in non-pore regions [33].

### 2.3 Long QT Syndrome Type 3 (LQT3)

LQT3 is less prevalent than LQT1 and LQT2, comprising approximately 5% to 10% of all LQTS cases [34]. It is primarily associated with mutations in the *SCN5A* gene, which encodes the cardiac sodium channel Na_v_1.5α subunit [35]. LQT3 often presents as pronounced bradycardia at rest. The likelihood of arrhythmic events increases with more pronounced QT interval prolongation at slower heart rates [36]. Gain-of-function *SCN5A* mutations cause LQT3 through multiple mechanisms: enhanced late sodium current, altered voltage-dependent gating, and channel trafficking defects [37]. Despite its lower incidence compared to LQT1 and LQT2, LQT3 is associated with the highest incidence of fatal cardiac events, particularly during periods of rest and sleep [38].

### 2.4 Other Special Types of LQTS

Conversely, LQT8, also referred to as Timothy syndrome (TS), is caused by multiple mutations in the *CACNA1C* gene, most notably the heterozygous c.1216G>A variant in exon 8A (p.G406R) in the α1 subunit of the L-type voltage-gated calcium channel Ca_V_1.2. Other pathogenic variants include exon 8 mutations (p.G406R, p.G402S) and mutations in alternative exons (p.Gly1911Arg, p.Arg518Cys), which result in phenotypic variability ranging from classic TS to isolated cardiac manifestations [39]. The Ca_V_1.2 channel is extensively expressed in both the developing and mature nervous systems, as well as in certain progenitor and glial cells, often resulting in multi-organ abnormalities [40]. Clinical manifestations of LQTS may encompass a prolonged QT interval, facial abnormalities such as small teeth and low-set ears, nervous system abnormalities including autism and epilepsy, epidermal abnormalities, and intermittent hypoglycemia [41,42,43,44]. Triadin (TRDN)–associated LQTS has been reported primarily in patients with homozygous or compound heterozygous truncating variants, often presenting with early-onset and severe arrhythmias [15]. Notably, TRDN mutations are more commonly associated with structural heart defects and overlapping phenotypes—including catecholaminergic polymorphic ventricular tachycardia (CPVT) and arrhythmogenic cardiomyopathy features with extensive T-wave inversions—rather than isolated LQTS [16]. Initial descriptions were based on a limited number of families, although subsequent registry-based studies have expanded the clinical spectrum of this entity. For completeness, it should also be noted that atrial arrhythmias, particularly atrial fibrillation, have been reported in association with congenital LQTS and other inherited channelopathies, as documented in the recent multicenter aTrial arrhythmias in inhEriTed aRrhythmIa Syndromes (TETRIS) study [45].

## 3. Conventional Management for LQTS

Despite recent advances in biologic therapies, current clinical management of congenital LQTS still relies predominantly on conventional strategies, including lifestyle modification, β-blockers [46], sodium-channel blockers [47], LCSD [7], and ICDs [48]. Contemporary evidence indicates that β-blockers [49], LCSD [50], and ICDs [51] can substantially reduce arrhythmic events and mortality in appropriately selected patients, although the magnitude of benefit varies across genotypes, risk groups, and study populations. With appropriate management, prognosis is substantially improved, and life expectancy may approach normal in many patients.

Lifestyle modification and trigger avoidance remain foundational for all patients, regardless of symptom status. These measures include avoiding QT-prolonging medications and promptly correcting electrolyte disturbances caused by conditions such as diarrhea or vomiting. In addition, preventive recommendations may vary by genotype: patients with LQT1 are generally advised to avoid strenuous or competitive exercise, whereas those with LQT2 should minimize exposure to sudden auditory stimuli and intense emotional stress.

### 3.1 Pharmacological Therapy

β-blockers, as the first-line therapy for LQTS, have demonstrated substantial efficacy, particularly in patients with the LQT1 subtype, consistent with the adrenergic-triggered arrhythmogenic mechanism characteristic of this genotype [49,52]. Previous research has indicated that β-blockers can reduce the risk of sudden cardiac death in LQTS patients; however, their protective effect varies across genotypes and does not eliminate the residual risk associated with persistent repolarization abnormalities [53]. Sodium channel blockers shorten the QT interval by selectively inhibiting the late sodium current (I_Na_-L) [54], thereby partially normalizing action potential duration without correcting mutant channel expression or trafficking abnormalities. Mexiletine, a typical late sodium current blocker, can shorten the QT interval without significantly affecting depolarization parameters [55]. Mexiletine was proposed as a genotype-directed therapy for patients with LQT3 and prolonged QTc as early as 1995, and has since shown clinically meaningful QT-shortening effects in selected patients [56,57]. Recent studies have also shown that mexiletine can shorten repolarization in patients with LQT2, patient-derived induced pluripotent stem cell (iPSC) models, and transgenic rabbit models [58,59]; however, this effect remains functional rather than corrective, as it does not restore normal hERG channel trafficking or expression.

### 3.2 Interventional and Device-Based Therapies

LCSD, a minimally invasive surgical procedure, decreases sympathetic nerve stimulation of the heart and is primarily indicated for patients who exhibit inadequate response to pharmacological treatment or are intolerant to β-blockers [60]. However, its efficacy varies across genotypes, and it does not directly modify the underlying ion channel dysfunction. A previous study has confirmed that LCSD can significantly reduce arrhythmia events in LQTS patients and that surgery-related complications are relatively few [61].

ICDs are predominantly employed as a therapeutic intervention for high-risk patients, particularly those who have experienced cardiac arrest or recurrent syncope unresponsive to pharmacological treatment [62]. Despite the efficacy of ICDs in managing arrhythmias, they are associated with certain complications, including inappropriate shocks, infections, and lead malfunctions [63]. These complications are especially pronounced in pediatric and young adult populations and may significantly affect their quality of life [51].

Collectively, these conventional therapies predominantly mitigate arrhythmic triggers or downstream consequences without correcting the primary molecular defects caused by pathogenic ion channel variants. These limitations underscore why molecularly targeted approaches are not merely adjunctive, but mechanistically necessary for the treatment of inherited long QT syndromes.

## 4. Progress in Biological Treatment of LQTS

Owing to the inherent limitations of conventional pharmacological therapies, there is a growing interest in exploring biological treatment strategies for LQTS, including gene therapy, antibody-based interventions, and other molecular approaches.

### 4.1 Gene Therapy

#### 4.1.1 Current Gene Therapy

The clinical application of gene therapy requires the efficient and accurate delivery of edited genetic sequences into target cells using appropriate vectors. These vectors can be broadly categorized into viral and non-viral systems. However, for inherited arrhythmia syndromes such as LQTS, effective and durable gene delivery to post-mitotic cardiomyocytes remains the principal translational constraint. Among them, the viral vectors mainly include adeno-associated viruses (AAVs) [64], adenoviruses [65] and lentiviruses [66]. Non-viral vectors mainly include lipid nanoparticles (LNPs) [67], plasmids [68], polymers [69], and nanoparticles [70].

AAVs are small, non-enveloped, single-stranded DNA viruses that require helper viruses (such as adenoviruses) for replication. AAV possesses a single-stranded DNA genome comprising two open reading frames (ORFs) that encode regulatory proteins and capsid antigens, with the genome flanked by inverted terminal repeats (ITRs). In AAV vectors, these ORFs are excised and substituted with expression cassettes designed for therapeutic proteins or RNA [71]. After injection, the antigens in the AAV vector capsid are present in the inoculum, and the single-stranded DNA is converted into double-stranded DNA in the cell nucleus, but unlike transgenic products, they are not actively produced by the transduced cells, so their genetic information will gradually be lost in mitotic cells. AAV vectors are widely used because of their relatively low immunogenicity, lack of self-replication, and efficient gene transfer. Nonetheless, AAV’s relatively limited packaging capacity, which restricts it from effectively packaging DNA exceeding 5 kb, presents a significant challenge in treating genetic disorders involving mutations in large genes [72].

Adenoviruses are non-enveloped double-stranded DNA (dsDNA) icosahedral viruses with a size of 90–100 nm. Adenoviral vectors can accommodate approximately 25–45 kb of DNA genomes [73]. Adenovirus became the first DNA virus to enter strict treatment development due to its clear biological definition, genetic stability, large transgenic capacity, and ease of large-scale production. Lentiviruses are spherical, enveloped, single-stranded RNA viruses with a diameter of roughly 100 nm [74]. Lentiviral particles encapsulate two positive-stranded RNAs that bind to nucleocapsid proteins. This particle also contains reverse transcriptase, integrase, and protease proteins. The entry of infectious lentiviral particles into host cells is mediated by the interaction between glycoproteins anchored on the outer envelope and specific cellular receptors. After successfully binding to the cell surface receptor, a series of reactions will be triggered, leading to the fusion of the viral particle lipid bilayer with the cell, and then the viral genetic material is released into the cytoplasm.

LNPs, with phospholipids as the main component, have a spontaneously formed closed spherical structure and are composed of one or more concentric curved lipid bilayer membranes and cholesterol [75]. Bacterial plasmids are usually small circular DNA elements outside the chromosome, capable of semi-autonomous replication through the mechanism of recruiting host cells. Plasmids typically do not encode essential genes required by the host but carry genes that may help the host adapt to the new environment [76]. Polymers can achieve safe and targeted drug delivery as well as sustained and controlled release of drugs through surface physical adsorption or chemical binding and internal structure inclusion of drugs [69]. Nanoparticles are granular dispersions or solid particles with a particle size ranging from 10 to 1000 nm [70]. They can be divided into organic nanoparticles and inorganic nanoparticles. Among them, organic nanoparticles include cationic polymer nanoparticles and lipid-based systems. Inorganic nanoparticles are mainly composed of inorganic particles and biodegradable polycations.

Overall, viral vectors can efficiently deliver genes, but they are also prone to inducing mutations, enhancing immunogenicity and inflammatory responses, and the production cost of viruses is relatively high. However, non-viral vectors allow easy large-scale production and chemical characterization, have higher loading capacity and transgenic ability, low toxicity, less immunogenicity and inflammatory response, and are safer, but their expression efficiency is relatively low. Therefore, when choosing a carrier, careful consideration is needed to select the most suitable one. To date, among available delivery platforms, cardiotropic AAV vectors—particularly AAV9—remain the most feasible option for *in vivo* cardiac gene delivery in LQTS. Proof-of-concept studies in LQT1 and LQT3 models (detailed in Section 5) have established AAV9-mediated gene replacement and base editing as promising strategies, whereas non-viral systems largely remain experimental due to insufficient efficiency in adult cardiomyocytes.

#### 4.1.2 Gene Editing

Gene-editing approaches in LQTS aim to correct or functionally compensate for pathogenic variants in a mutation-mechanism-dependent manner. In LQTS, the feasibility of gene editing depends strongly on the underlying mutation mechanism; gain-of-function variants such as those in SCN5A may be more amenable to targeted correction than dominant-negative loss-of-function variants, which often require allele-specific strategies. Key techniques employed in gene therapy include CRISPR (clustered regularly interspaced short palindromic repeats)/Cas9 gene editing [77], cytosine base editor (CBE) [78], adenine base editor (ABE) [79] and prime editing (PE) [80] (Fig. 3). CRISPR/Cas9, an RNA-guided gene editing tool, functions as an RNA-mediated adaptive immune defense system. Cas9, part of the type II CRISPR system, utilizes a guide RNA (gRNA) to bind to target DNA via base pairing, facilitating DNA cleavage. Subsequent cellular repair mechanisms, such as nonhomologous end joining (NHEJ) or homology-directed repair (HDR), enable gene knockout or insertion [81]. This technology is predominantly applied in gene knockout, regulation of endogenous gene expression, live cell labeling of chromosome loci, single-stranded RNA editing, and high-throughput gene screening, offering advantages of cost-effectiveness, flexibility, and ease of use. Nonetheless, CRISPR/Cas9 technology can induce DNA double-strand breaks, potentially resulting in insertions and deletions (indels) or non-specific editing. Especially in LQTS-causing dominant-negative mutations, such as many KCNQ1 and KCNH2 variants, allele-specific suppression combined with preservation of wild-type channel function is often required, substantially increasing technical complexity and off-target risk. Consequently, concerns persist regarding the safety and efficacy of CRISPR’s delivery mechanisms.

**Fig. 3.**
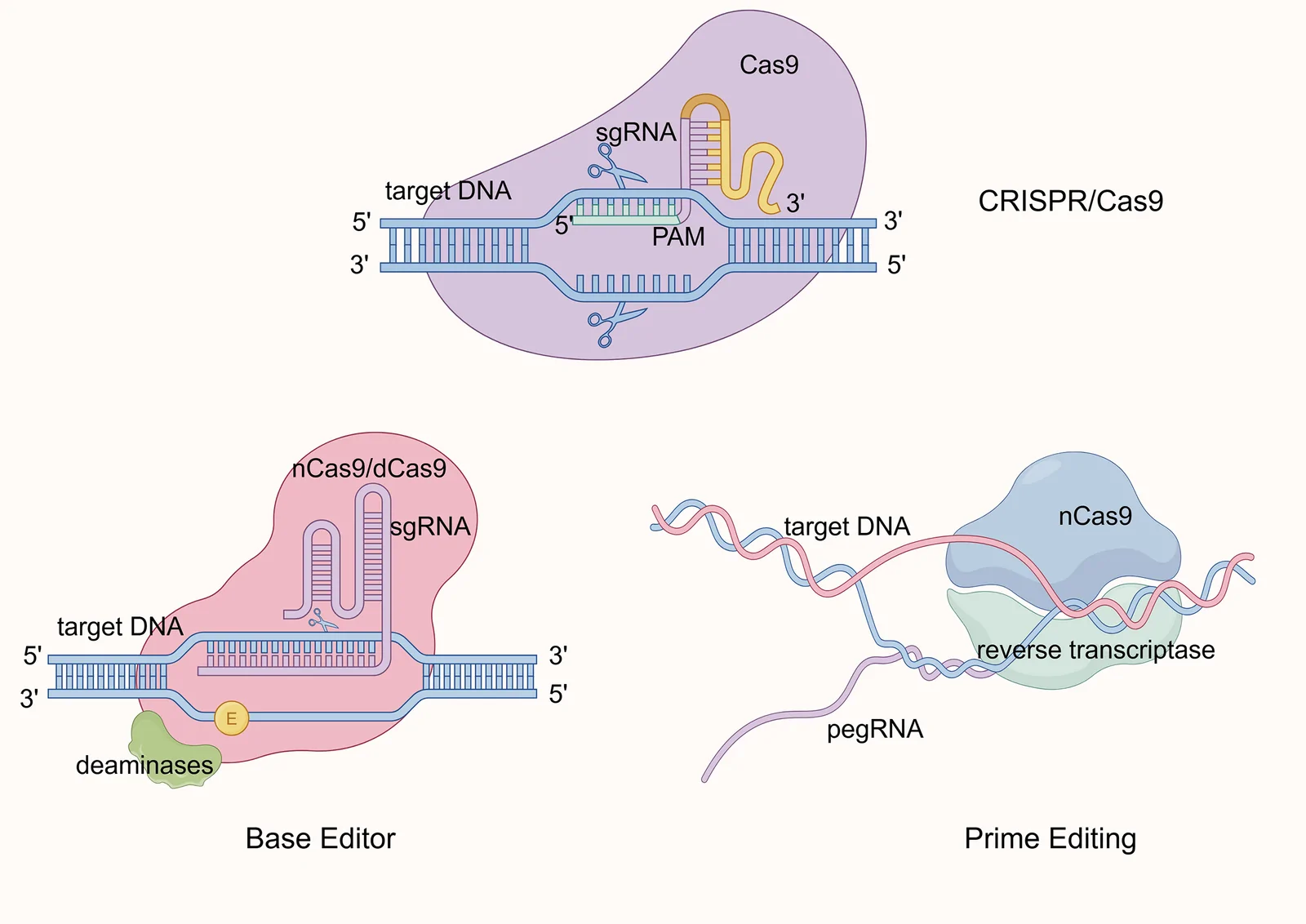
**Comparison of gene editing strategies**. CRISPR/Cas9: sgRNA brings Cas9 to the target site containing PAM (NGG), promoting double-stranded DNA cleavage. Base editor: Modified Cas proteins (such as nCas9 or dCas9) are fused with a deaminase to create a fusion protein that binds the target DNA sequence under the guidance of gRNA. The Cas component then nicks one DNA strand, exposing the target base for chemical conversion. Prime editing: Cas9 H840 nickase nicks the target DNA strand adjacent to the PAM sequence. The exposed 3′ end of the nicked DNA strand anneals and binds to the 3′ primer binding site (PBS) sequence of pegRNA. Reverse transcriptase then extends the edited DNA using the RT template encoded in the pegRNA. CRISPR, clustered regularly interspaced short palindromic repeats; PAM, protospacer adjacent motif; NGG, N followed by two guanines / canonical SpCas9 PAM sequence; pegRNA, prime editing guide RNA; gRNA, guide RNA; RT, reverse transcriptase.

To address these challenges, researchers have investigated alternative gene editing technologies that do not involve double-strand breaks, such as CBE, ABE, and PE. The base editor is formed by fusing modified Cas proteins (such as nCas9 or dCas9) with deaminases (which can directly modify the chemical structure of DNA bases) to create fusion proteins. It binds to the target DNA sequence under the guidance of gRNA. Subsequently, the Cas portion (nCas9) in the fusion protein will cut one strand of the DNA at the target location (single-strand break), or simply bind but not cut (dCas9), thereby exposing the target base. Finally, the deaminase directly acts on the exposed target base, leading to base conversion. Therefore, base editors can directly convert a single base without causing double-strand breaks in DNA, avoiding potential genomic instability and improving editing accuracy.

Although cytosine and adenine base editors can efficiently mediate four types of base conversions without inducing double-strand breaks, they are currently incapable of addressing the remaining eight base substitutions or enabling insertions and deletions. To overcome these limitations, David Liu’s laboratory developed prime editing in 2019, based on the CRISPR/Cas9 platform [80]. In this system, a Cas9 nickase variant (nCas9-H840A) is fused to a reverse transcriptase, forming a novel fusion protein. Simultaneously, a specialized RNA molecule—prime editing guide RNA (pegRNA)—is engineered by appending an extended RNA sequence to the 3′ end of a conventional single-guide RNA (sgRNA). This additional sequence fulfills two distinct functions: one segment acts as a primer binding site (PBS), annealing to the 3′ end of the nicked DNA strand to initiate reverse transcription, while the other serves as a template encoding the desired point mutation or insertion/deletion. This dual-function design allows PE to achieve precise and versatile genome edits without generating DSBs.

Qi et al. [82] developed a dual AAV9-ABE delivery system, which has been successfully applied to a LQT3 mouse model harboring the SCN5A gene p.T1307M mutation. In this system, the Cas9 protein and the gRNA/adenine deaminase components of ABE are divided into two separate AAV9 vectors. Functional restoration is achieved through *in vivo* recombination, effectively eliminating pathogenicity while preserving normal channel function. In LQTS, the feasibility of gene editing depends on the mutation mechanism. For dominant-negative KCNQ1 mutations, allele-specific suppression is required to preserve wild-type channel function. In contrast, gain-of-function SCN5A mutations are amenable to direct correction: Qi et al. [82] achieved 99.2% editing efficiency using dual AAV9-ABE in an LQT3 mouse model. These studies illustrate the genotype-specific considerations for applying gene editing in LQTS.

#### 4.1.3 Gene Silencing

Gene-silencing strategies aim to reduce the expression of mutant proteins by inhibiting translation of mutant transcripts or promoting mRNA degradation, and are particularly relevant to diseases caused by dominant-negative mutations. The technical means of gene silencing mainly include CRISPR interference (CRISPRi), small interfering RNA (siRNA), short hairpin RNA (shRNA), and Antisense oligonucleotides (ASO).

CRISPRi mainly utilizes dCas9 to precisely bind to the promoter region or coding sequence of the target gene under the guidance of sgRNA, hindering the binding of transcription factors and the function of RNA polymerase, thereby inhibiting gene transcription [83]. As for the three types of small nucleic acid drugs with silencing effects, namely siRNA, shRNA, and ASO, they will be discussed in the following text. In general, gene therapy can work at the genetic level and can be applied to ion channel diseases with clear pathogenic genes. Although gene-silencing strategies are theoretically attractive for dominant-negative LQTS mutations, their clinical translation is limited by allele specificity requirements, delivery efficiency, and the need for lifelong cardiac suppression.

### 4.2 RNA Interference

RNA interference technology suppresses the expression of specific genes via siRNA, shRNA, or ASO, thereby mitigating the detrimental effects of mutant genes. siRNA is a double-stranded small RNA [84]. It binds to the mRNA of target genes and inhibits their translation for a relatively short period of time. Due to its short duration of action, long-term administration is required. shRNA is a single-stranded RNA sequence with a tight hairpin turn. It mainly expresses itself continuously and inhibits the expression of target genes after transfection into target cells, and has the characteristics of long-term expression and stable silencing [85]. An ASO is a single-stranded oligonucleotide molecule (18–30 nt), which can be DNA, RNA or a hybrid single-stranded DNA/RNA [86]. After entering cells, it can complement target genes and cause mRNA degradation under the action of ribonuclease H1 (RNase H1), thereby inhibiting protein expression. Alternatively, through steric hindrance, ASOs can modulate pre-mRNA splicing to regulate gene translation, thereby achieving therapeutic effects.

The selective targeting of mutant alleles using allele-specific siRNA has demonstrated promise as a therapeutic approach for various cardiac syndromes, proving to be safe, effective, and feasible [87,88]. At present, mature siRNA drugs have been applied in the clinical treatment of hypercholesterolemia. However, unlike hepatic targets, efficient and sustained delivery of siRNA to cardiomyocytes remains a major unresolved challenge, limiting direct extrapolation to LQTS. Nonetheless, for diseases characterized by multiple related mutations, this mutation-specific strategy is both labor-intensive and costly.

### 4.3 Antibody Therapy

Compared with gene- and RNA-based approaches, antibody-based strategies for LQTS remain highly exploratory and are currently supported mainly by cellular and animal proof-of-concept studies. Antibody-based proteins serve as crucial biological therapeutic agents due to their intrinsic capacity to bind antigens and endogenous immune receptors, coupled with the stability, specificity, and adaptability inherent in the antibody framework [89]. The inaugural therapeutic monoclonal antibody, muromonab-CD3 (OKT3), received approval from the United States Food and Drug Administration (FDA) in 1985 [90]. The fundamental role of an antibody is to neutralize target antigens. This is achieved by binding to overlapping epitopes or inducing conformational alterations, thereby inhibiting the interaction between receptors and their co-receptors or growth factors. Beyond neutralization, antibodies possess the ability to cross-link, leading to the precipitation of soluble antigens or the agglutination of cells. Currently, innovative antibody derivatives have superseded conventional monoclonal antibodies, offering solutions to the intricate pathobiology of diseases and enhancing existing therapeutic interventions. Srinivasan conducted a study in which they developed high-affinity nanobodies capable of specifically recognizing Na_V_1.4 (skeletal muscle) and Na_v_1.5 (myocardial muscle). They also validated the therapeutic potential of these nanobodies, thereby offering a novel approach for the diagnosis and treatment of cardiac ion channel disorders [91].

Jervell–Lange–Nielsen syndrome (JLNS) is an autosomal-recessive form of congenital LQTS most commonly caused by biallelic *KCNQ1* or *KCNE1* mutations that reduce I_Ks_ and are associated with congenital sensorineural hearing loss. Although JLNS is mechanistically distinct from hERG-related disease, recent work using hiPSC–derived cardiomyocytes (hiPSC-CMs) showed that a PAS-targeting hERG1 activator (scFv2.10) increased I_Kr_ currents, shortened APD, and reduced arrhythmic events, thereby illustrating the broader therapeutic potential of channel-activating antibody fragments in severe repolarization disorders [92]. Li et al. [93] produced anti-KCNQ1 antibodies by injecting *KCNQ1* channel peptides into rabbits. It was found that rabbits vaccinated with the *KCNQ1* vaccine showed a shortened QTc, but the durability of this effect and its safety profile remain unclear: the study did not determine whether QT correction was permanent or required maintained antibody titers, and the risk of excessive QT shortening or autoimmune complications warrants caution when translating this approach to therapeutic monoclonal antibodies.

The technologies underlying LQTS biologic therapies have progressed to human application: AAV gene therapy is approved for spinal muscular atrophy [94]; CRISPR-based base editing is in trials for familial hypercholesterolemia [95]; RNAi drugs (inclisiran) are established for cardiovascular disease [96] and immunotherapies for autoimmune neurologic channelopathies (anti-NMDAR encephalitis) provide precedent for treating excitable cell disorders with biologic agents [97]. These precedents support the broader translational feasibility of biologic therapies, although substantial disease-specific challenges remain for LQTS. The overview of LQTS biological treatment is shown in Fig. 4.

**Fig. 4.**
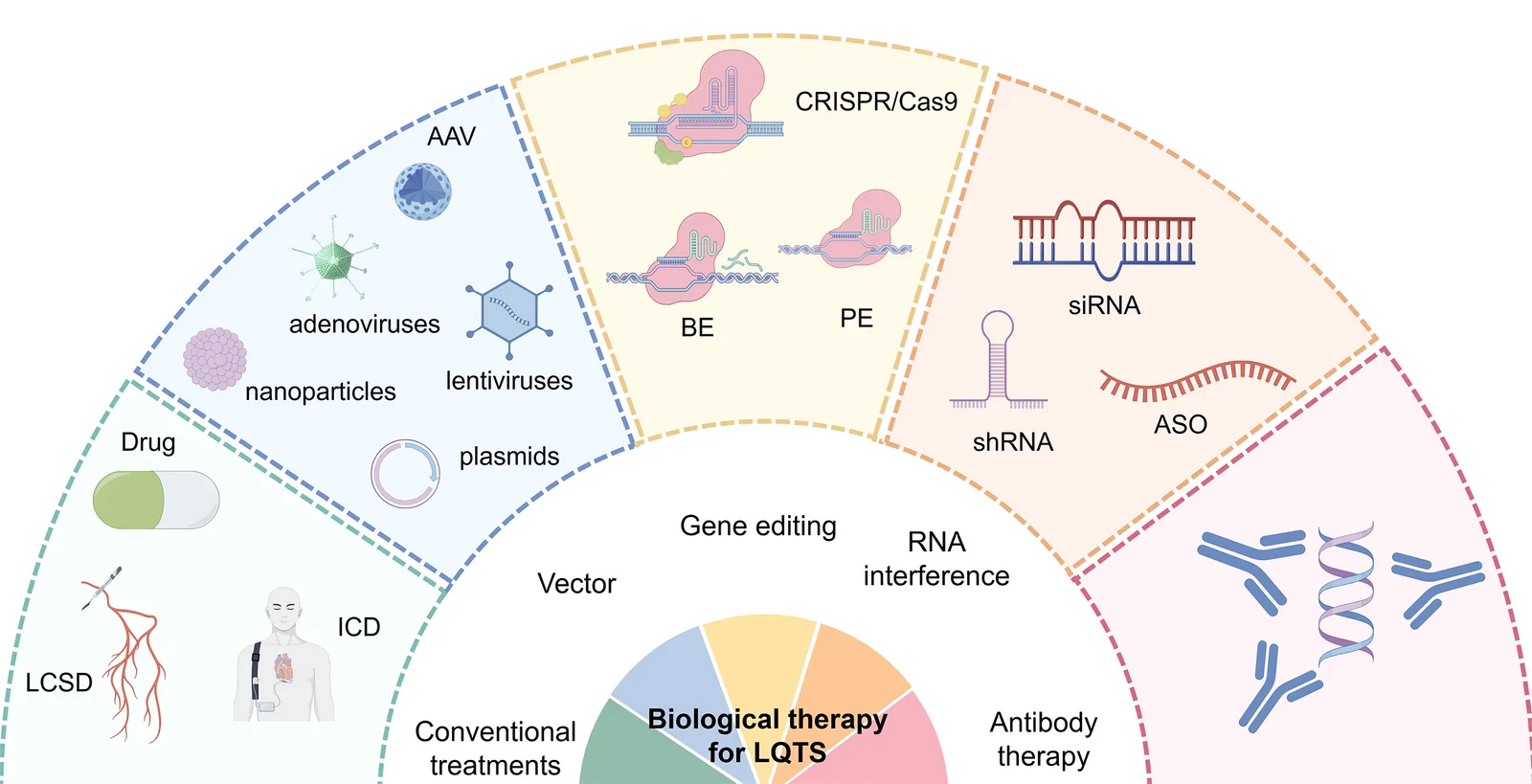
**Treatment of long QT syndrome**. Current treatments for LQTS include conventional drug and surgical treatments, as well as emerging molecular approaches such as gene editing, RNA interference, and antibody-based therapy. LCSD, left cardiac sympathetic denervation; ICD, implantable cardioverter-defibrillator; AAV, adeno-associated viruses; CRISPR, clustered regularly interspaced short palindromic repeats; BE, Base Editing; PE, Prime Editing; siRNA, small interfering RNA; shRNA, short hairpin RNA; ASO, Antisense oligonucleotides.

## 5. Emerging Biological Therapies Across LQTS Subtypes

Because treatment responsiveness differs substantially across genotypes, emerging biologic strategies should be interpreted in the context of subtype-specific clinical management. Beyond general clinical management, an increasing number of studies are exploring personalized biological therapies tailored to distinct forms of long QT syndrome. These approaches vary according to the underlying disease mechanism, with some subtypes being more amenable to direct mutation correction, others to modulation of ion-channel expression or trafficking, and still others to RNA-based intervention. Therefore, evaluation of these emerging therapies requires not only consideration of their molecular targets, but also careful integration with genotype-specific pathophysiology and clinical characteristics.

In a recent study, researchers employed an ABE system delivered via dual adeno-associated virus (AAV) in a mouse model to address the LQT3 phenotype associated with the p.T1307M mutation, achieving an editing efficiency of up to 99.2% at the transcriptional level in cardiac tissue. This study provided proof-of-concept that *in vivo* base editing could correct a pathogenic SCN5A variant and improve the LQT3 phenotype in mice [82]. Earlier proof-of-concept studies also showed that augmentation of repolarizing potassium currents, such as adenoviral delivery of wild-type Kv1.5 in transgenic mice with a long-QT phenotype, could shorten APD and QT interval; however, this strategy is not mutation-specific and differs conceptually from contemporary precision gene-editing approaches [98]. Furthermore, the aforementioned ASO can modulate exon 8A in a dose- and time-dependent manner, thereby restoring ion flux dynamics, calcium dynamics, and associated cell motility defects, offering a novel therapeutic strategy for long QT syndrome type 8 (LQT8) [40].

Giannetti et al. [99] investigated the effects of varying concentrations of serum and glucocorticoid regulated kinase-1 (SGK1) inhibitors in hiPSC-CMs, and transgenic rabbit models of LQT1 and LQT2. Their findings indicated that SGK1 inhibitors consistently shortened the APD in LQT2 across different variants and species, while the effects in the LQT1 model varied depending on the specific variants [99]. These findings suggest that SGK1 inhibition may have broader efficacy in LQT2 than in LQT1, where treatment response appears to be more gene- and variant-specific. Previous research has demonstrated that SGK1 inhibitors reduce APD in LQT3 hiPSC-CMs [100]. Furthermore, Limpitikul et al. [101] developed an iPSC line carrying the CALM2-D130G mutation and employed CRISPRi to selectively silence the expression of mutant genes. This approach functionally restored the Ca^2+^/CaM-dependent inactivation of L-type calcium channels and the morphology of cardiac action potentials, supporting the feasibility of mutation-directed precision approaches for selected forms of LQTS.

Additionally, Li et al. [102] identified the agonist-like anti-KCNQ1 autoantibodies present in the sera of a subset of patients with dilated cardiomyopathy. Specifically, these autoantibodies enhance the K_v_7.1 channel current (I_Ks_) in HEK293 cells expressing the *KCNQ1*/*KCNE1* gene by targeting the channel’s pore region. Furthermore, the findings indicated that the reduction in APD and QT interval was attributed to the enhanced I_Ks_ in rabbit cardiomyocytes immunized with *KCNQ1* channel peptides. Kis and Li [103] employed hybridoma technology to generate mouse monoclonal antibodies, demonstrating that these antibodies could normalize the duration of cardiac action potentials in hiPSC-CMs in a pharmacological model of LQT3 and suppress arrhythmias. Additionally, the research conducted by the Maguy team [104] revealed that antibodies generated from peptides derived from the pore region of the *KCNQ1* channel could intrinsically increase I_Ks_ density, thereby shortening the APD and QT interval, and potentially serve as a therapeutic approach for LQT2. Besides, Yamamoto et al. [105] rescued the electrophysiological abnormalities of LQT15 hiPSC-CMs by using the CRISPR-Cas9 system for specific knockout of mutant alleles. These findings provide proof-of-concept for allele-specific genome editing as a potential therapeutic strategy for calmodulin-associated LQTS.

Pan et al. [106] developed a series of computational models, including single-cell, one-dimensional, and three-dimensional cardiac conduction models, to simulate the electrophysiological alterations associated with various genotypes (wild-type, heterozygous, homozygous). They assessed the impact of *KCNQ1* antibodies on parameters such as the QT interval and repolarization dispersion. The findings indicated that while *KCNQ1* antibodies have the potential to shorten the QT interval, they may concurrently increase repolarization dispersion, thereby elevating the risk of arrhythmias. These antibodies have demonstrated the capacity to modulate ion channel currents in both cellular and animal models, suggesting their potential utility in correcting electrocardiographic phenotypes. Nonetheless, further investigation is required to determine the optimal dosage of these antibodies and to elucidate their effects on arrhythmia biomarkers at the tissue and organ levels. Table 2 (Ref. [40,72,82,92,99,101]) lists representative preclinical studies that support biological treatment strategies for LQTS.

**Table 2.** **Representative preclinical studies supporting emerging molecular and biologic strategies for LQTS**.

Model level	Gene/subtype	Intervention	Key outcome	Translational status	Reference
Mouse	*SCN5A* (T1307M)	*In vivo* base editing (ABE, dual AAV9)	Corrected pathogenic allele; QT/APD normalized	Proof-of-concept; off-target/safety pending	Qi et al. [82]
Rabbit	*KCNQ1* (Y315S)	AAV9 suppression–replacement gene therapy	Restored I_Ks_, normalized QT/APD	Large-animal Proof-of-concept; long-term safety required	Bains et al. [72]
hiPSC	*CACNA1C* (G406R) (Timothy/LQT8)	ASO regulating exon 8A splicing	Reversed pathological electrophysiology/morphology	Proof-of-concept	Chen et al. [40]
hiPSC-CMs	*CALM2* (D130G)	CRISPRi allele-specific silencing	Restored Ca^2+^ handling and APD	Proof-of-concept (*in vitro* only)	Limpitikul et al. [101]
hiPSC-CMs	*KCNQ1* (Trp188X) (JLN)	scFv2.10 (PAS-targeted antibody fragment)	Increased I_Kr_, shortened APD, reduced EADs	Proof-of-concept	Ukachukwu et al. [92]
hiPSC-CMs; cardiac cell sheets; rabbit	Broad (LQT1/2)	SGK1 inhibitors (small molecules)	Shortened APD across genotypes	Proof-of-concept preclinical; Candidate compounds in translational pipeline	Giannetti et al. [99]

## 6. Future Directions and Research Gaps

Although biological therapies for arrhythmias remain in the early stages of development, they hold substantial promise for genetically well-characterized ion channelopathies. The continued evolution of omics technologies and the growing emphasis on precision medicine are expected to drive the transition toward genotype-guided treatment strategies for LQTS. Techniques such as gene editing, RNA interference, and antibody therapies offer the potential to personalize interventions based on individual genetic profiles, ultimately improving treatment efficacy and reducing adverse effects. Nevertheless, significant challenges remain. The delivery of therapeutic agents to cardiac tissues is a major obstacle, particularly for gene editing technologies. Long-term expression stability, immunogenicity of viral vectors (especially AAVs), and off-target effects must be addressed. Lentiviral vectors, while effective, carry a risk of insertional mutagenesis. Moreover, gene editing strategies are currently best suited for single-point mutations and may not be broadly applicable across all LQTS subtypes. Thus, further research is needed to develop more universal therapeutic approaches.

Bridging the gap between basic research and clinical application is critical for advancing biological therapies from bench to bedside. Multidisciplinary collaboration among molecular biologists, electrophysiologists, pharmacologists, and bioengineers will be essential to overcoming these challenges and translating experimental advances into tangible clinical outcomes.

Future research should therefore focus on improving cardiac delivery platforms, optimizing allele-specific and mutation-specific approaches, and validating therapeutic efficacy in clinically relevant models. Large-animal studies, long-term safety assessments, and standardized arrhythmia endpoints will be essential for bridging the gap between proof-of-concept studies and clinical application. Ultimately, addressing these challenges will be critical for realizing the promise of precision biological therapies for LQTS. Fig. 5 summarizes the current treatment landscape and remaining translational challenges of LQTS.

**Fig. 5.**
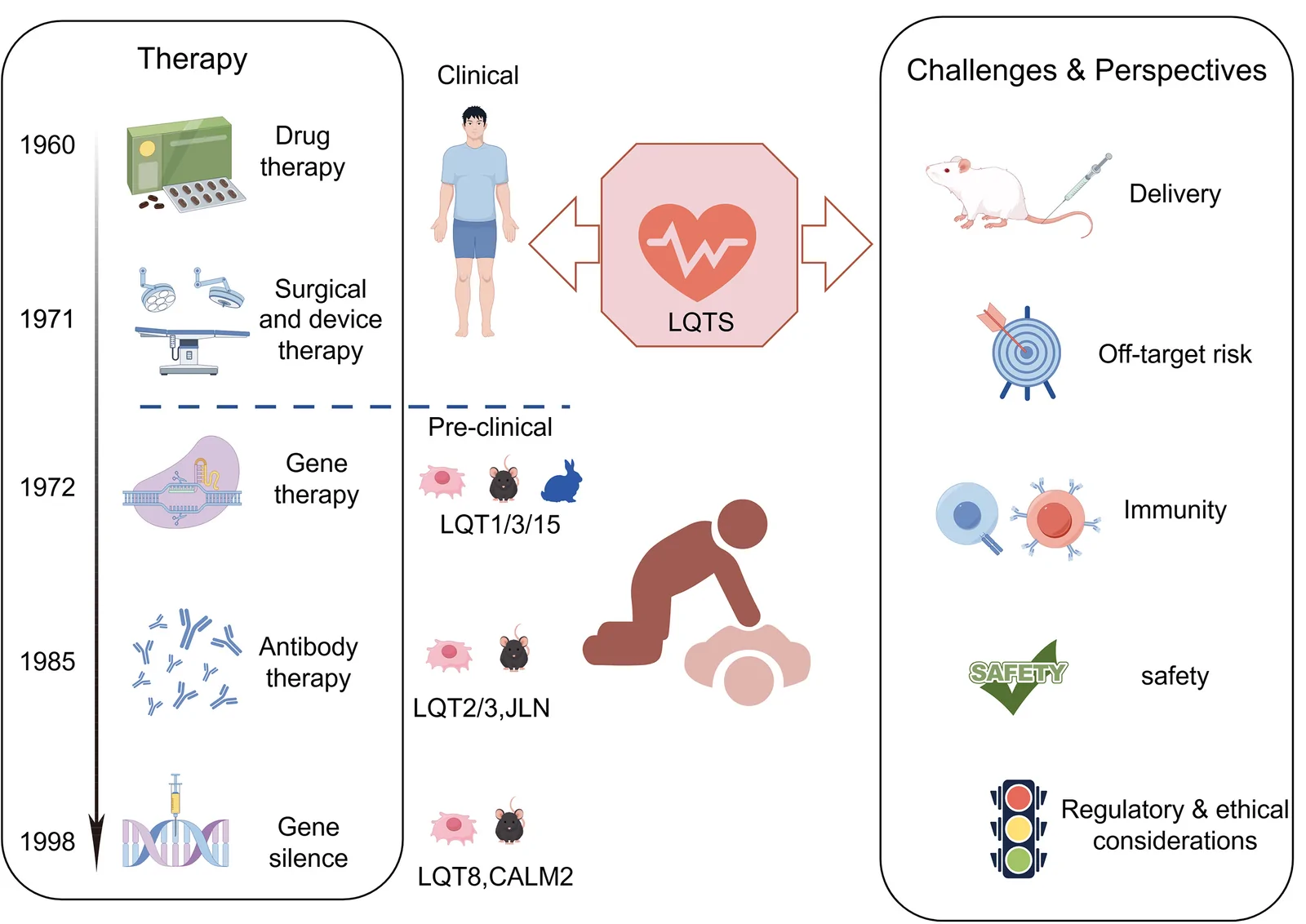
**Overview of the treatment and challenges of LQTS**. The left section summarizes the therapeutic landscape for LQTS. The middle-left column details the existing treatment strategies, with the upper portion indicating therapies already in clinical use, and the lower portion highlighting those under preclinical investigation—including the vectors utilized and the LQTS subtypes studied. The middle-right section illustrates the clinical manifestations of the syndrome. The right side of the overview presents the remaining challenges in LQTS management. JLN, Jervell–Lange–Nielsen.

## 7. Limitations

This review has several limitations. First, the rapidly evolving nature of biologic therapies for LQTS means that the preclinical studies discussed herein represent a snapshot in time, and clinical translation timelines remain uncertain. Second, the genetic heterogeneity of LQTS-with multiple genes implicated but a limited subset having definitive or strong evidence-limits the generalizability of mutation-specific therapies. Finally, this review did not systematically assess cost-effectiveness or health equity considerations, which will be critical for the accessibility of these emerging therapies.

## 8. Conclusions

Long QT syndrome is a prototypical cardiac ion channelopathy for which conventional therapies reduce risk but do not correct the underlying molecular defects. Emerging biologic approaches, including gene editing, RNA interference, and antibody-based strategies, have shown promising preclinical potential to address these limitations. However, their clinical translation will require more robust validation, improved cardiac delivery, and careful evaluation of long-term safety. Overall, continued advances in molecular therapeutics and interdisciplinary collaboration will be essential to realizing precision medicine for LQTS.
